# Exercise Training Has Contrasting Effects in Myocardial Infarction and Pressure Overload Due to Divergent Endothelial Nitric Oxide Synthase Regulation

**DOI:** 10.3390/ijms19071968

**Published:** 2018-07-06

**Authors:** Elza D. van Deel, Yanti Octavia, Monique C. de Waard, Martine de Boer, Dirk J. Duncker

**Affiliations:** 1 Division of Experimental Cardiology, Department of Cardiology, Thoraxcenter, Erasmus MC, University Medical Center Rotterdam, 3015GD Rotterdam, The Netherlands; e.vandeel@erasmusmc.nl (E.D.v.D.); octaviayanti.md@gmail.com (Y.O.); m.dewaard@erasmusmc.nl (M.C.d.W.); g.deboer@erasmusmc.nl (M.d.B.); 2 Department of Cardiology, Cardiovascular Research Institute Maastricht, Maastricht University Medical Centre, 6229ER Maastricht, The Netherlands

**Keywords:** exercise, myocardial infarction, aortic stenosis, oxidative stress, nitric oxide synthase

## Abstract

The beneficial effects of exercise training (EX) on cardiac pathology are well recognized. Previously, we found that the effects of EX on cardiac dysfunction in mice critically depend on the underlying etiology. EX exerted beneficial effects after myocardial infarction (MI); however, cardiac pathology following pressure overload produced by transverse aortic constriction (TAC) was aggravated by EX. In the presented study, we investigated whether the contrasting effects of EX on cardiac dysfunction can be explained by an etiology-specific response of endothelial nitric oxide (NO) synthase (eNOS) to EX, which divergently affects the balance between nitric oxide and superoxide. For this purpose, mice were exposed to eight weeks of voluntary wheel running or sedentary housing (SED), immediately after sham, MI, or TAC surgery. Left ventricular (LV) function was assessed using echocardiography and hemodynamic measurements. EX ameliorated LV dysfunction and remodeling after MI, but not following TAC, in which EX even aggravated fibrosis. Strikingly, EX attenuated superoxide levels after MI, but exacerbated NOS-dependent superoxide levels following TAC. Similarly, elevated eNOS S-glutathionylation and eNOS monomerization, which were observed in both MI and TAC, were corrected by EX in MI, but aggravated by EX after TAC. Additionally, EX reduced antioxidant activity in TAC, while it was maintained following EX in MI. In conclusion, the present study shows that EX mitigates cardiac dysfunction after MI, likely by attenuating eNOS uncoupling-mediated oxidative stress, whereas EX tends to aggravate cardiac dysfunction following TAC, likely due to exacerbating eNOS-mediated oxidative stress.

## 1. Introduction

During the last decade, heart failure (HF) has developed into a major public-health burden worldwide [1,2]. Consequently, the development of novel strategies for the treatment of HF becomes increasingly important. In addition to optimal pharmacotherapy, physical exercise training (EX) is increasingly implemented in the rehabilitation process of HF patients. The beneficial effects of EX on exercise capacity and cardiovascular performance are well established [3], and translate into improvements in quality of life, as well as reductions in HF-related hospitalizations and mortality [3,4,5,6,7].

An important player in the many protective effects of exercise is endothelial nitric oxide (NO) synthase (eNOS) [8,9,10]. In the presence of its co-factors, eNOS transfers electrons from reduced nicotinamide adenine dinucleotide phosphate (NADPH), via the flavin adenine dinucleotide and flavin mononucleotide to the heme site, where electrons are utilized to reduce and activate oxygen, and to oxidize L-arginine to L-citrulline and NO [11]. EX increases shear stress and mechanical stretch, which stimulate eNOS activity and increase NO production [9,12]. NO has various beneficial effects on cardiovascular function, including vasodilation, the inhibition of platelet aggregation and adhesion, the inhibition of leukocytes and vascular inflammation, the stimulation of angiogenesis, the proliferation of vascular smooth muscle cells, and the activation of endothelial progenitor cells [13]. In addition to the stimulation of eNOS, EX beneficially influences the cardiac nitroso–redox balance by activating reactive oxygen species (ROS) scavenger enzymes such as superoxide dismutases (SODs) [14,15]. Accordingly, we and others have demonstrated that EX improves survival and attenuates LV dysfunction, oxidative stress, and fibrosis in a mouse model of myocardial infarction (MI) [16,17,18,19], and have additionally demonstrated that full eNOS expression is required for these beneficial EX effects after MI [20].

In contrast to experimental [18] and clinical [21] evidence demonstrating beneficial effects of EX after MI, patients with obstruction of the left ventricular (LV) outflow tract, are advised to perform only mild exercise or to refrain from physical strain [22,23]. In line with these guidelines, we previously found that EX failed to exert a beneficial effect, and even tended to aggravated cardiac dysfunction and remodeling in mice suffering from chronic pressure overload produced by transverse aortic constriction (TAC) [24].

The mechanism underlying these contrasting effects of EX in different cardiac pathologies remains unresolved, but could involve divergent effects of EX on eNOS. Under pathological conditions, eNOS can uncouple and convert from an NO− to a superoxide (O_2_^−^)-producing enzyme. Several mechanisms have been proposed to induce eNOS uncoupling, including eNOS monomerization [13] and S-glutathionylation of eNOS [25]. Uncoupled eNOS is still capable of transferring electrons, but the electrons are diverted to oxygen rather than to L-arginine, resulting in the production of O_2_^−^, and subsequently, further eNOS uncoupling [13].

In light of these considerations, we hypothesized that EX influences the nitroso–redox balance and eNOS in a pathology-specific manner so that EX can either stimulate NO or eNOS-mediated superoxide production, depending on the underlying cardiac pathology. To test our hypothesis, we investigated how EX affects eNOS and the nitroso–redox balance in two distinct but clinically highly relevant cardiac pathologies by studying the effects of EX in mice with either MI or TAC.

## 2. Results

### 2.1. Exercise and Survival

**Figure 1:**
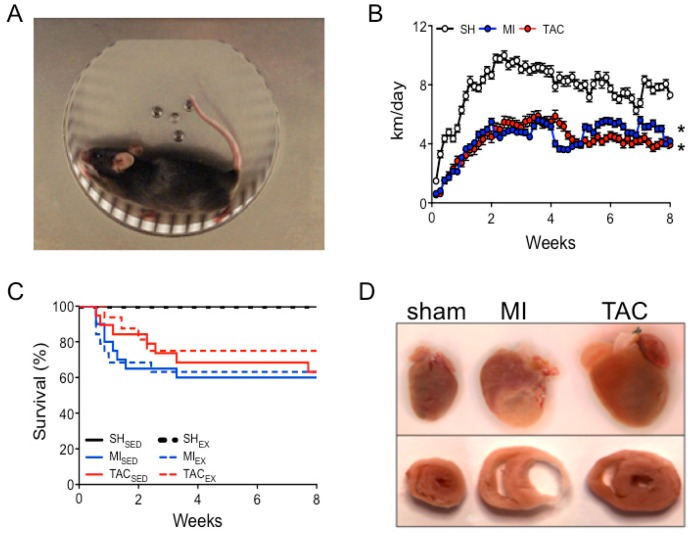
Experimental models, daily running distance, and survival. (**A**) Voluntary wheel running; (**B**) daily running distance of sham (SH), myocardial infarction (MI), and transverse aortic constriction (TAC) mice; (**C**) Kaplan–Meier survival curve for all groups; (**D**) post-mortem examples of hearts and cardiac cross sections of sham mice and mice with an MI or TAC. Total number of animals entering the study in voluntary running (EX) and sedentary housing (SED) groups: SH_SED_ (*n* = 12), SH_EX_ (*n* = 22), MI_SED_ (*n* = 21), MI_EX_ (*n* = 19), TAC_SED_ (*n* = 19), and TAC_EX_ (*n* = 16). * *p* < 0.05 total running distance vs. total running distance of sham mice.

All mice in the EX groups began running voluntarily on the first day after surgery (Figure 1A,B). The total eight-week distance was lower in TAC_EX_ (237 ± 57 km) and MI_EX_ (245 ± 47 km) than in sham (SH)_EX_ mice (435 ± 60 km; Figure 1B). In the sedentary housing (SED) group, MI_SED_ and TAC_SED_ had survival rates of 62% and 63%, respectively, while EX did not affect mortality in any of the groups (Figure 1C).

### 2.2. LV Remodeling and Dysfunction

**Figure 2:**
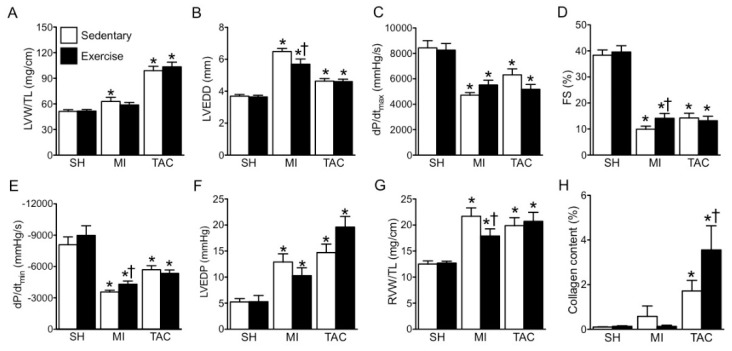

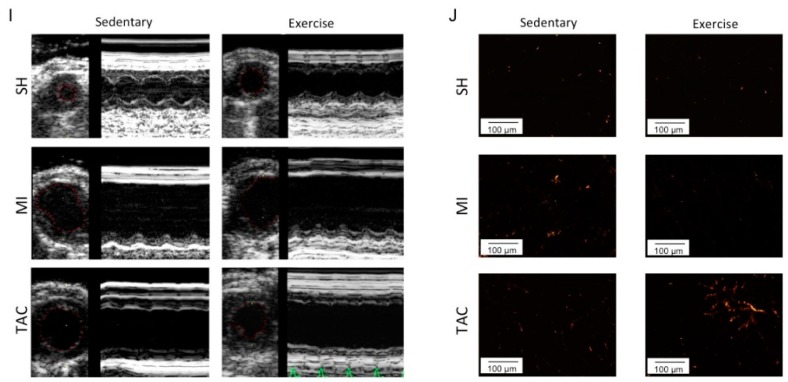
Exercise (EX) mitigates cardiac remodeling and dysfunction following myocardial infarction (MI), but not after transverse aortic constriction (TAC). (**A**) Left ventricle (LV) weight (LVW) per tibia length (TL); (**B**) LV end diastolic lumen diameter (LVEDD); (**C**) maximum rate of rise in LV pressure (dP/dt_max_); (**D**) fractional shortening (FS); (**E**) maximum rate of fall in LV pressure (dP/dt_min_); (**F**) LV end diastolic pressure (LVEDP); (**G**) right ventricle weight (RVW) per TL; (**H**) collagen content; (**I**) representative LV short axis and M-Mode echo images; (**J**) representative images of collagen staining. * *p* < 0.05 vs. corresponding sham, † *p* < 0.05 vs. corresponding sedentary. *n* = 12 in all groups.

MI and TAC both increased relative LV weight and lumen diameter (Figure 1D and Figure 2A,B), decreased LV systolic dysfunction, as indicated by LV dP/dt_max_ and fractional shortening (FS), as well as early (LV dP/dt_min_) and late LV diastolic dysfunction (LV end diastolic pressure (LVEDP)) (Figure 2C–F,I). Furthermore, in both MI and TAC, cardiac backward failure was evidenced by an increase in relative right ventricular (RV) weight (Figure 2G), while marked LV fibrosis was observed in TAC (Figure 2H,J). EX improved LV remodeling and dysfunction after MI. In contrast, EX failed to ameliorate, and even tended to aggravate LV remodeling and dysfunction after TAC (Figure 2).

### 2.3. Superoxide Production

**Figure 3:**
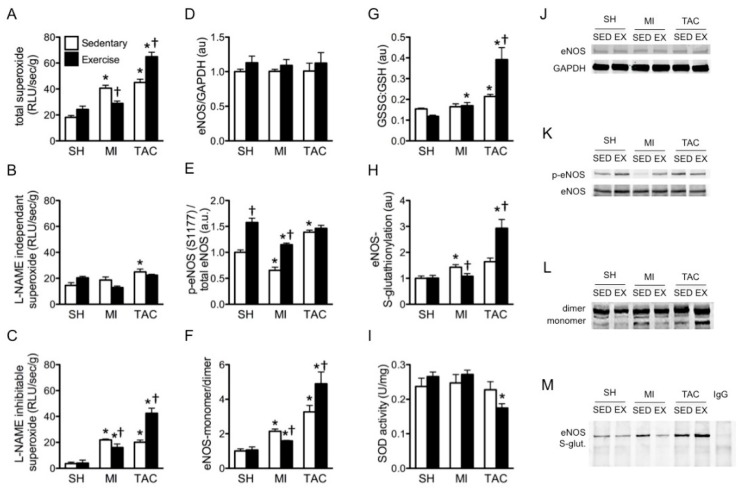
Assessment of exercise (EX) effects on superoxide, endothelial nitric oxide synthase (eNOS), and glutathionylation after sham (SH), myocardial infarction (MI), and transverse aortic constriction (TAC). (**A**) Lucigenin-enhanced chemiluminescence superoxide production is expressed as relative light unit (RLU) per second per gram protein; (**B**) evaluation of superoxide production with N^G^-nitro-L-arginine methyl ester (L-NAME), presented as L-NAME-independent superoxide; (**C**) subtraction of L-NAME-independent superoxide from total superoxide, presented as L-NAME-inhibitable superoxide; (**D**) eNOS protein expression; (**E**) phosphorylated eNOS (p-eNOS) serine (Ser)1177 protein expression; (**F**) eNOS monomer–dimer ratio; (**G**) oxidized glutathion (GSSG) and reduced glutathion (GSH) ratio; (**H**) S-glutathionylation of eNOS; (**I**) superoxide dismutase (SOD) activity; (**J**) representative western blot of eNOS protein expression; (**K**) representative western blot of p-eNOS serine (Ser)1177 protein expression; (**L**) representative eNOS monomer dimer blot; (**I**) representative co-immunoprecipitation of eNOS S-glutathionylation with unspecific mouse immunoglobulin G (IgG) antibody as a negative control. p-eNOS: phosphorylated eNOS; eNOS S-glut: eNOS S-glutathionylation. * *p* < 0.05 vs. corresponding sham, † *p* < 0.05 vs. corresponding sedentary. *n* = 3–8 in all groups.

Having established that the effects of exercise on LV function and remodeling were etiology-dependent, we sought to determine the potential role of etiology-dependent superoxide generation in the effects of exercise. In LV myocardium of the sedentary groups, MI and TAC both increased superoxide generation compared to sham-operated animals (Figure 3A). This increase was abolished in MI and markedly reduced in TAC by NOS inhibition with N^G^-nitro-L-arginine methyl ester (L-NAME; Figure 3B). Interestingly, EX inhibited superoxide production in MI, but significantly increased superoxide levels following TAC (Figure 3A). Similarly, L-NAME-inhibitable, and therefore, NOS-dependent superoxide generation was blunted by EX following MI, but markedly aggravated by EX following TAC (Figure 3C). These findings suggest a direct functional relation between NOS and the contrasting effects of EX in myocardial superoxide production.

### 2.4. Total eNOS and Phosphorylated eNOS (p-eNOS) Protein Expression

To investigate whether the divergent effects of EX in MI and TAC could be explained by eNOS expression or phosphorylation levels, we examined the effect of EX on eNOS protein expression and eNOS serine (Ser)1177 phosphorylation. There was no difference in total cardiac eNOS protein expression between sham, MI, and TAC groups either with or without EX (Figure 3D,J). In contrast, p-eNOS Ser1177 levels were reduced after MI, but were increased after TAC. EX significantly increased the expression of p-eNOS Ser1177 in sham and MI groups, but did not further change p-eNOS Ser1177 levels in TAC mice (Figure 3E,K).

### 2.5. eNOS Uncoupling and eNOS S-Glutathionylation

Since eNOS uncoupling is a potential inducer of eNOS-mediated superoxide formation, we subsequently determined the eNOS monomer–dimer ratio and the level of eNOS S-glutathionylation. The eNOS monomer–dimer ratio was increased after both MI and TAC, and reduced by EX in MI, but further elevated by EX in TAC mice (Figure 3F,L).

The oxidized glutathione disulfide (GSSG) to reduced glutathione (GSH) ratio was not affected by MI, but was elevated after TAC. Additionally, the increase in superoxide levels in TAC_EX_ mice was accompanied by a further increase in the GSSG:GSH ratio (Figure 3G). Moreover, while S-glutathionylation of eNOS was modestly elevated in MI and TAC mice, this increase in eNOS S-glutathionylation was normalized by EX in MI, but aggravated in exercised TAC mice (Figure 3H,M).

Taken together, eNOS S-glutathionylation and eNOS monomerization data indicate that eNOS uncoupling is normalized by EX following MI, but aggravated by EX in the presence of TAC.

### 2.6. Superoxide Dismutase (SOD) Activity

To investigate whether divergent effects of EX on eNOS-mediated superoxide generation additionally involved altered antioxidant activity, we studied the activity of SOD. Total SOD activity was not changed after MI or TAC compared to sham-operated animals. Interestingly, EX did not alter SOD activity in sham and MI groups, but significantly decreased SOD activity in TAC mice (Figure 3I).

## 3. Discussion

**Figure 4:**
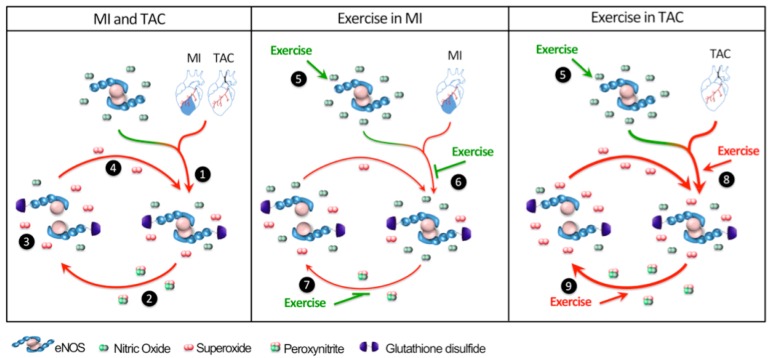
Divergent effects of exercise (EX) on endothelial nitric oxide synthase (eNOS) regulation after myocardial infarction (MI) and transverse aortic constriction (TAC). MI and TAC induce eNOS S-glutathionylation (1), resulting in eNOS uncoupling and eNOS-mediated superoxide production (2). The consequently formed peroxynitrite further uncouples eNOS by inducing eNOS monomerization and more eNOS-mediated superoxide production (3), which further uncouples eNOS (4). EX stimulates eNOS (5). Beneficial effects of EX following MI: EX diminishes eNOS S-glutathionylation (6), as well as eNOS monomerization (7). Detrimental effects of EX following TAC: EX aggravates eNOS S-glutathionylation (8), thus inducing more eNOS monomerization (9), resulting in further elevation of superoxide production.

In the presented study, we tested the hypothesis that eNOS-derived NO and superoxide play a key role in the divergent effects of EX on cardiac remodeling and dysfunction following MI versus chronic pressure overload produced by TAC. The major findings were that (i) eight weeks of voluntary EX after MI decreased superoxide production, whereas EX following TAC increased superoxide production; (ii) these divergent responses were principally explained by EX-induced reduction of eNOS-mediated myocardial superoxide levels after MI, as opposed to an EX-induced increase in eNOS-mediated superoxide production in TAC; (iii) accordingly, EX reduced eNOS uncoupling, evidenced by reductions in myocardial eNOS S-glutathionylation and eNOS monomerization after MI, whereas EX aggravated TAC-induced eNOS uncoupling evidenced by increased S-glutathionylation and eNOS monomerization after TAC (Figure 4). 

EX is known to produce multiple cardiac changes, including physiological cardiac hypertrophy, increased SOD activity, and reduced oxidative stress [26]. However, the EX levels of healthy individuals are not easily achieved by patients with severe cardiac disease, which is why we subjected our mice to a voluntary wheel-running protocol. This likely explains why our sham-operated animals demonstrated only trends toward the cardiac adaptations reported for intense EX training. Still, the EX protocol did produce skeletal muscle adaptations as evidenced by increased citrate synthase activity in skeletal muscle [17]. Moreover, the mild-to-moderate EX levels performed in our study significantly affected the diseased heart by stimulating cardiac performance after MI, while aggravating the nitroso–redox imbalance in the heart following TAC. In the current study, the beneficial effects of eight weeks of EX on cardiac function were not accompanied by improved survival. This might be partly explained by the fact that mortality in C57Bl6 MI mice is largely caused by cardiac rupture [27], and EX started after the onset of MI does not influence this phenomenon [28]. To study the long-term effects of EX on survival after MI and TAC, it would be interesting to study exercising MI and TAC mice for a longer period of time.

One of the key components of the beneficial effects of EX in cardiovascular disease is improved endothelial function through eNOS [12,29]. Accordingly, previous studies from our laboratory showed that the beneficial effects of EX following MI require full eNOS expression [20]. In line with this observation, eNOS overexpression was shown to improve cardiac function after MI [30,31]. In the present study, we found that exercise after MI attenuated eNOS uncoupling, which explains the reduction of eNOS-dependent superoxide production after EX in MI.

It is known that divergent cardiac pathologies activate distinct pathological signaling pathways that result in specific disease characteristics [32]. Consequently, measures that prove to be protective of the diseased heart are not necessarily equally beneficial in all cardiac etiologies. Accordingly, although the benefit of EX is clearly established in most forms of cardiac disease [7], patients with moderate-to-severe aortic stenosis are recommended to avoid competitive sports to reduce the risk of sudden death [22,23]. We recently observed that EX not only failed to ameliorate, but even tended to aggravate cardiac dysfunction in chronic pressure overload produced by TAC [24]. In the present study, we found that this lack of benefit from EX in TAC is accompanied by EX-induced worsening of eNOS uncoupling (evidenced by eNOS S-glutathionylation and eNOS monomerization), as well as EX-induced reduction of SOD activity, leading to increased eNOS-mediated oxidative stress. Even though we did not demonstrate a direct causal relationship between elevated ROS and aggravated cardiac dysfunction following TAC and EX, the data strongly suggest that elevated eNOS-mediated ROS levels importantly contribute to the worsening of cardiac performance when TAC is combined with EX. Likely, the fixed stenosis in TAC counteracts an important part of the beneficial effects of eNOS since it precludes the reduction in LV afterload resulting from eNOS-mediated systemic vasodilation. Indeed, beneficial effects of EX were described in hypertension, where stimulation of eNOS is able to reduce cardiac afterload [33,34]. Moreover, the fixed stenosis amplifies the EX-induced increase in LV systolic pressure, and hence, LV afterload during each exercise bout [35]. A meta-analysis performed by Haykowsky et al. [36] suggests this may adversely affect the LV response to EX by showing that dynamic EX, which results in modest increases in LV systolic pressure, had beneficial effects on ejection fraction in heart-failure patients, whereas static EX, which is known to result in more marked elevations in LV systolic pressure, failed to improve ejection fraction [11]. Apparently, the EX-induced increase in LV systolic pressure during EX in TAC exacerbated the TAC-induced cardiac pathology, and further aggravated detrimental eNOS uncoupling and concomitant oxidative stress. Treatment of TAC mice with phosphodiesterase type 5 (PDE5) inhibitors was previously shown to improve cardiac pathological hypertrophy [37]. Therefore, it could be interesting to explore whether treatment of EX TAC mice with NO donors might mitigate the detrimental effects of exercise and TAC on eNOS uncoupling and consequent ROS production. However, this should be the topic of future studies.

Clinical guidelines recommend at least 30 min of physical activity on most, if not all, days of the week to reduce risk factors for chronic disease [38,39]. In cardiovascular disease patients, EX not only improves exercise performance and quality of life, but also reduces morbidity and mortality [3,5,6,7]. Notwithstanding these clear benefits of EX, EX restriction is recommended for patients with aortic stenosis [22]. However, no beneficial effects of EX restriction were observed in a large cohort study involving more than 500 patients suffering from congenital aortic stenosis [40]. Thus, the potential benefits or hazards of EX in patients with aortic stenosis remain under debate, and recommendations appear to be based on pathophysiological considerations, rather than on hard clinical evidence [7]. Our study shows that, unlike after MI, EX may aggravate cardiac pathology in the presence of an aortic stenosis because of an etiology-dependent effect of EX on the nitroso–redox balance. We demonstrate that the balance between cardiac NO and superoxide is differentially affected by EX in diverse etiologies and pivotal in the mechanisms of both beneficial and detrimental effects of EX.

In conclusion, the presented study demonstrates that the balance between cardiac NO and superoxide plays a pivotal role in determining both the beneficial and detrimental effects of EX. Thus, the underlying pathology determines whether eNOS tilts the nitroso–redox balance toward either a beneficial or a detrimental influence. This observation may be important for therapies aiming to restore the nitroso–redox balance in patients with heart failure due to different etiologies, and may aid in improving disease-mechanism-specific interventions.

## 4. Materials and Methods

All experiments were performed in accordance with and following approval by the Animal Research Committee of the Erasmus MC University of Rotterdam (27 June 2006 nr: 109-06-12 EUR976). A total of 99 C57Bl/6 mice 12–20-week-old mice entered the study: sham sedentary (SH_SED_; *n* = 12), sham exercise (SH_EX_; *n* = 12), MI sedentary (MI_SED_; *n* = 21), MI exercise (MI_EX_; *n* = 19), TAC sedentary (TAC_SED_; *n* = 19), and TAC exercise (TAC_EX_; *n* = 16). Males and females were equally distributed between groups. 

### 4.1. Animal Experiments

**Figure 5:**
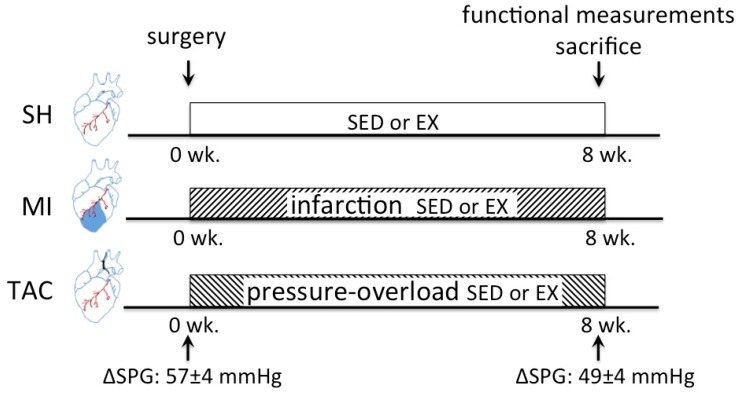
Timeline of SH, MI, and TAC experiments. wk., week; SED, sedentary; EX, exercise; ΔSPG, systolic pressure gradient. (ΔSPGs are historical data from van Deel et al. [24]).

All mice were sedated with 4% isoflurane, intubated, and connected to a pressure-controlled ventilator (SAR-830/P; CWE, Ardmore, PA, USA). Anesthesia was maintained using 2.5% isoflurane, while body temperature was kept at 37 °C, and buprenorphine was administered (50 μg/kg; intraperitoneally (i.p.)) for postsurgical analgesia. Sham, MI, and TAC surgery were performed as previously described [24,41]. After recovery from surgery, all exercise groups were exposed to eight weeks of voluntary wheel running (Figure 1B). The timeline of SH, MI, and TAC experiments is visualized in Figure 5.

### 4.2. Cardiac Function and Geometry Measurements

Eight weeks after sham, MI, or TAC surgery, all mice were re-anesthetized and ventilated, and body temperature was kept at 37 °C while M-mode LV echocardiography was performed using an Aloka SSD 4000 echo device (Aloka; Tokyo, Japan). Following echocardiography, a pressure catheter (Millar Instrument; Houston, TX, USA) was inserted into the LV via the carotid artery, and LV pressure (LVP) was measured. LV dP/dt_max_ and LV dP/dt_min_ were later calculated using MatLab [24]. Subsequently, the heart was excised and the LV, right ventricle (RV), and lung weight, as well as tibia length, were measured. LV tissue was rapidly snap-frozen after extraction, and stored for further molecular analysis, or embedded in paraffin for histological analysis. In MI animals, all analyses were performed in remote, viable, LV myocardium.

### 4.3. Histology

Paraffin-embedded LV tissue of six mice per group was serially sectioned (4 µm). Collagen content was measured after Picrosirius red staining under polarized light, and analyzed with a quantitative image-analysis system (Clemex Technologies, Longueuil, QC J4G 1T5, Canada).

### 4.4. Detection of Superoxide Production

Superoxide generation in homogenized snap-frozen LV tissue (4–6 samples per group) was evaluated by lucigenin-enhanced chemiluminescence using a luminometer (Luminoskan Ascent, Thermo Fisher Scientific, Waltham, MA, USA)) as previously described [42]. Briefly, LV tissue was homogenized in Krebs-Hepes buffer, dark-adapted lucigenin (final concentration 5 µM) was added to the homogenates, and basal chemiluminescence was measured after reaching equilibrium. Subsequently, NOS-dependent superoxide production was measured by repeating the measurements after adding the NOS inhibitor, N^G^-nitro-L-arginine methyl ester (L-NAME; 1 mM), to the homogenates. Light emission was recorded, and was expressed as relative light units (RLU)/sec/g. Each experiment was performed in duplicate.

### 4.5. Total and Phosphorylated eNOS Protein Analysis

Total eNOS and p-eNOS protein levels were determined in LV homogenates as previously described [43], and normalized to glyceraldehyde 3-phosphate dehydrogenase (GAPDH; Novus Biologicals, Littleton, CO, USA)).

Subsequent to SDS-PAGE, the proteins were transferred to nitrocellulose membranes, and the blots were probed with primary anti-eNOS (1:500, BD Transduction Laboratory, San Jose, CA, USA), anti-p-eNOS (1:1000 Cell Signaling, Danvers, MA, USA), anti-GAPDH (1:10,000, Imgenex), and secondary rabbit anti-mouse immunoglobulin G (IgG) antibody conjugated with horseradish peroxidase (HRP; 1:1000, Santa Cruz Biotechnology, Dallas, TX, USA). All blots were analyzed using the Odyssey system (LI-COR, Lincoln, NE 68504, USA).

### 4.6. Quantification of GSSG and GSH

The oxidized glutathione disulfide (GSSG) to reduced glutathione (GSH) ratio was assessed using a glutathione assay kit (Cayman Chemical, Ann Arbor, MI, USA). LV tissue was homogenized in cold 2-(N-morpholino) ethanesulfonic acid (MES) buffer, before being centrifuged at 10,000× *g* for 15 min at 4 °C. Samples were deproteinated using metaphosporic acid and triethanolamine (Sigma-Aldrich, Saint Lois, MI, USA), and total GSH was immediately measured at 405 nm at five-minute intervals for 30 min. GSSG was measured independently, using 2-vinylpiridine (Sigma-Aldrich).

### 4.7. eNOS S-Glutathionylation

To determine S-glutathionylation of eNOS, an eNOS pull-down assay was performed with a protein G-conjugated (Novex) anti-eNOS antibody (Santa Cruz). Subsequently, immunoblotting was performed using an anti-glutathione monoclonal antibody (ViroGen, Watertown, MA, USA). To confirm the detection of eNOS S-glutathionylation, control samples were treated with dithiothreitol, which removes the glutathionylation modification of eNOS.

### 4.8. eNOS Monomer–Dimer Ratio

The eNOS monomer–dimer ratio was determined using low-temperature SDS-PAGE (LT-PAGE) as previously described [43]. Briefly, total proteins were incubated in 5× Laemmli buffer without 2-mercaptoethanol, and the gels and buffers were equilibrated at 4 °C before electrophoresis, during which the buffer tank was placed in an ice bath to maintain the temperature of the gel. After probing with the anti-eNOS antibody (BD Transduction Laboratory), immunoreactive bands were visualized and analyzed with the Odyssey system (LI-COR).

### 4.9. SOD Activity

To evaluate the effects of EX on the SOD activity, intracellular SOD activity (cytosolic (SOD1) and mitochondrial (SOD2)) activity was measured using a commercially available kit (Cayman chemical).

### 4.10. Statistical Analysis

Data are expressed as means ± standard error of the mean (SEM). Statistical significance was assessed using a two-way ANOVA, followed by post-hoc testing with Newman–Keuls test. Total running distances were compared with one-way ANOVA, followed by Newman–Keuls post-hoc test. A value of *p* < 0.05 was considered as statistically significant (two-tailed).

Similar to previous observations [24], we did not observe an influence of sex on the effects of TAC and MI and/or EX on the responses of survival and LV hypertrophy or dysfunction. Consequently, we pooled male and female mice for final analysis.
